# De novo variants in genes regulating stress granule assembly associate with neurodevelopmental disorders

**DOI:** 10.1126/sciadv.abo7112

**Published:** 2022-08-17

**Authors:** Xiangbin Jia, Shujie Zhang, Senwei Tan, Bing Du, Mei He, Haisong Qin, Jia Chen, Xinyu Duan, Jingsi Luo, Fei Chen, Luping Ouyang, Jian Wang, Guodong Chen, Bin Yu, Ge Zhang, Zimin Zhang, Yongqing Lyu, Yi Huang, Jian Jiao, Jin Yun (Helen) Chen, Kathryn J. Swoboda, Emanuele Agolini, Antonio Novelli, Chiara Leoni, Giuseppe Zampino, Gerarda Cappuccio, Nicola Brunetti-Pierri, Benedicte Gerard, Emmanuelle Ginglinger, Julie Richer, Hugh McMillan, Alexandre White-Brown, Kendra Hoekzema, Raphael A. Bernier, Evangeline C. Kurtz-Nelson, Rachel K. Earl, Claartje Meddens, Marielle Alders, Meredith Fuchs, Roseline Caumes, Perrine Brunelle, Thomas Smol, Ryan Kuehl, Debra-Lynn Day-Salvatore, Kristin G. Monaghan, Michelle M. Morrow, Evan E. Eichler, Zhengmao Hu, Ling Yuan, Jieqiong Tan, Kun Xia, Yiping Shen, Hui Guo

**Affiliations:** ^1^ Center for Medical Genetics and Hunan Key Laboratory of Medical Genetics, School of Life Sciences, Central South University; Changsha, Hunan 410078, China.; ^2^ Genetic and Metabolic Central Laboratory, Birth Defects Prevention and Control Institute of Guangxi Zhuang Autonomous Region, Maternal and Child Health Hospital of Guangxi Zhuang Autonomous Region, Nanning 530003, China.; ^3^ Department of Medical Genetics and Molecular Diagnostic Laboratory, Shanghai Children’s Medical Center, Shanghai Jiao Tong University School of Medicine, Shanghai 200000, China.; ^4^ NHC Key Laboratory of Birth Defect for Research and Prevention, Hunan Provincial Maternal and Child Health Care Hospital, Hunan, China.; ^5^ Department of Pediatrics, Daping Hospital, Army Medical University, Chongqing, China.; ^6^ Mental Health Center, West China Hospital of Sichuan University, Chengdu 610000, China.; ^7^ Massachusetts General Hospital Neurogenetics Unit, Department of Neurology, Massachusetts General Brigham, Boston, MA 02114, USA.; ^8^ Center for Genomic Medicine, Department of Neurology, Massachusetts General Hospital, Harvard Medical School, Boston, MA 02115, USA.; ^9^ Laboratory of Medical Genetics, Bambino Gesù Children’s Hospital, IRCCS, Rome 00165, Italy.; ^10^ Center for Rare Diseases and Birth Defects, Department of Woman and Child Health and Public Health, Fondazione Policlinico Universitario A. Gemelli-IRCCS, Rome 00168, Italy.; ^11^ Faculty of Medicine and Surgery, Catholic University of the Sacred Heart, Rome 00168, Italy.; ^12^ Fondazione Policlinico Universitario Agostino Gemelli Dipartimento Scienze della Salute della Donna e del Bambino, Rome, Italy.; ^13^ Università Cattolica S. Cuore, Dipartimento Scienze della Vita e Sanità Pubblica, Rome, Italy.; ^14^ Telethon Institute of Genetics and Medicine (TIGEM), Pozzuoli, Italy.; ^15^ Department of Translational Medicine, Federico II University, Naples, Italy.; ^16^ Institut de Génétique Médicale d’Alsace (IGMA), Laboratoire de Diagnostic Génétique, Hôpitaux universitaires de Strasbourg, Strasbourg, Alsace, France.; ^17^ Service de Génétique, Centre Hospitalier de Mulhouse, Mulhouse, Alsace, France.; ^18^ Department of Medical Genetics, Children’s Hospital of Eastern Ontario, Ottawa, Ontario, Canada.; ^19^ Department of Pediatrics, Neurology and Neurosurgery, Montreal Children’s Hospital, McGill University, Montreal, Canada.; ^20^ Children’s Hospital of Eastern Ontario Research Institute, University of Ottawa, Ottawa, Ontario, Canada.; ^21^ Department of Genome Sciences, University of Washington School of Medicine, Seattle, WA 98195, USA.; ^22^ Department of Psychiatry and Behavioral Sciences, University of Washington, Seattle, WA 98195, USA.; ^23^ Department of Pediatrics, Indiana University School of Medicine, Indianapolis, IN 46202, USA.; ^24^ Amsterdam University Medical Center, Department of Clinical Genetics, Amsterdam, Netherlands.; ^25^ University Medical Center Utrecht, Department of Paediatrics, Utrecht, Netherlands.; ^26^ Department of Human Genetics, Amsterdam Reproduction and Development Research Institute, Amsterdam University Medical Center, University of Amsterdam, Amsterdam, Netherlands.; ^27^ Pediatrics and Genetics, Alpharetta, GA 30005, USA.; ^28^ CHU Lille, Clinique de Génétique, Guy Fontaine, F-59000 Lille, France.; ^29^ Institut de Génétique Médicale, Université de Lille, ULR7364 RADEME, CHU Lille, F-59000 Lille, France.; ^30^ Department of Medical Genetics and Genomic Medicine, Saint Peter’s University Hospital, New Brunswick, NJ 08901, USA.; ^31^ GeneDx, Gaithersburg, MD 20877, USA.; ^32^ Howard Hughes Medical Institute, University of Washington, Seattle, WA 98195, USA.; ^33^ CAS Center for Excellence in Brain Science and Intelligences Technology (CEBSIT), Chinese Academy of Sciences, Shanghai 200000, China.; ^34^ Hengyang Medical School, University of South China, Hengyang, China.; ^35^ Division of Genetics and Genomics, Boston Children’s Hospital, Harvard Medical School, Boston, MA 02115, USA.; ^36^ Hunan Key Laboratory of Animal Models for Human Diseases, Changsha, Hunan 410078, China.

## Abstract

Stress granules (SGs) are cytoplasmic assemblies in response to a variety of stressors. We report a new neurodevelopmental disorder (NDD) with common features of language problems, intellectual disability, and behavioral issues caused by de novo likely gene-disruptive variants in *UBAP2L*, which encodes an essential regulator of SG assembly. *Ubap2l* haploinsufficiency in mouse led to social and cognitive impairments accompanied by disrupted neurogenesis and reduced SG formation during early brain development. On the basis of data from 40,853 individuals with NDDs, we report a nominally significant excess of de novo variants within 29 genes that are not implicated in NDDs, including 3 essential genes (*G3BP1*, *G3BP2*, and *UBAP2L*) in the core SG interaction network. We validated that NDD-related de novo variants in newly implicated and known NDD genes, such as *CAPRIN1*, disrupt the interaction of the core SG network and interfere with SG formation. Together, our findings suggest the common SG pathology in NDDs.

## Teaser

De novo mutations of stress granule genes are associated with neurodevelopmental disorders.

## INTRODUCTION

Neurodevelopmental disorders (NDDs) are a group of multifactorial conditions with overlapping symptoms, strong genetic predisposition, and shared risk genes (*1*), especially between intellectual disability (ID)/developmental delay (DD) and autism spectrum disorder (ASD). Recent large-scale exome or genome sequencing studies have achieved substantial progress in deciphering the genetic components underlying NDDs (*2*–*4*). However, the etiology and pathogenesis of a considerable proportion of NDD individuals are still elusive.

During early brain development, the coordinated expressions of many genes are strictly regulated in a spatially and temporally appropriate manner (*5*–*8*). Perturbance of gene expression regulation in early brain development affects normal developmental trajectory, which might lead to various NDDs (*8*–*10*). The fact that the majority of high-confidence NDD genes are classified as of function in gene expression regulation (*1*, *2*, *11*) further supported this gene expression dysregulation hypothesis of NDD. Stress granules (SGs) are membraneless compartments in eukaryotic cells that are dynamically induced upon environmental stresses (*12*, *13*). They play important roles in the regulation of gene expression (*14*–*16*). Although the mechanisms regarding SG dynamics are still largely elusive, hundreds of SG components and regulators (hereafter referred to as SG genes) have been identified (*13*, *17*, *18*). However, the association between variants in SG genes and NDDs is not well characterized.

In this study, conducted by a large-scale international collaboration, we revealed that de novo heterozygous likely gene-disruptive (LGD) variants in *UBAP2L*, a critical gene regulating SG formation, lead to a new NDD. *Ubap2l* haploinsufficiency in mouse leads to social disability and cognitive impairments, which resemble the symptoms that we observed in individuals with *UBAP2L* LGD variants. *Ubap2l* deficiency in mouse disrupts neurogenesis and interferes with SG formation in early brain development, supporting the association of SG defect with NDDs. Furthermore, the statistical data based on 40,853 individuals with NDDs and the subsequent functional evidence supported that de novo mutations (DNMs) in the essential SG genes, such as *G3BP1*, *G3BP2*, and *CAPRIN1*, are associated with NDD risk. In addition, we also provide a list of 26 newly implicated NDD genes, which are SG constituents or regulatory components for future studies.

## RESULTS

### Disruptive variants in *UBAP2L* lead to a new NDD

**Fig. 1.**
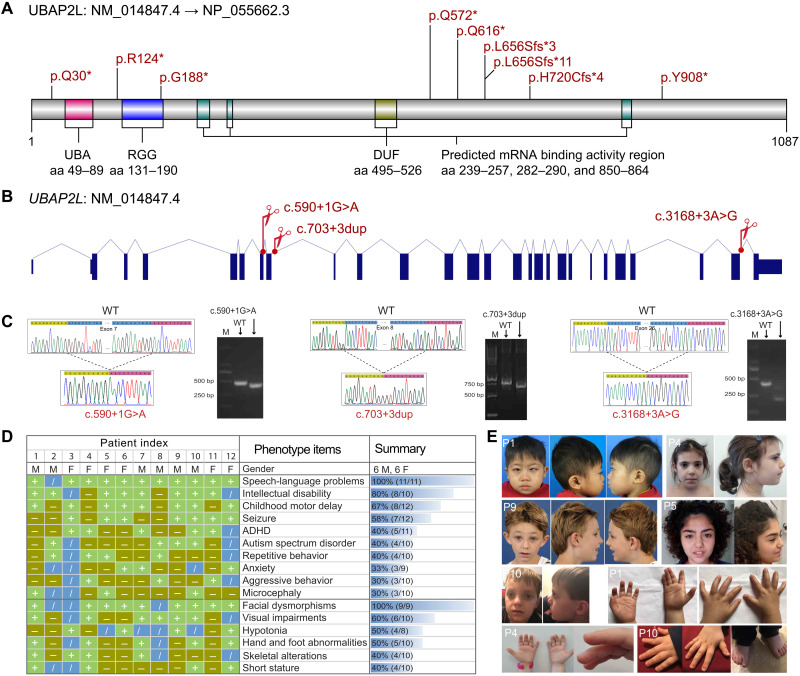
Disruptive variants in *UBAP2L* lead to a new NDD. (**A**) Distribution of de novo nonsense and frameshift variants in *UBAP2L* identified in NDDs is shown in a protein model. aa, amino acids. (**B**) Distribution of de novo splicing variants in *UBAP2L* identified in NDDs is shown in a gene model. (**C**) Minigene assay shows that de novo variants c.590+1G>A, c.703+3dup, and c.3168+3A>G in *UBAP2L* impair normal splicing. Sanger sequencing confirmed that the variants resulted in skipping of exons 7, 8, and 26, respectively, which are highlighted by blue. M represents marker. Sequence highlighted by green above the Sanger trace is from exon 25. Sequence highlighted by pink is from exon 27. (**D**) Phenotypic spectrum of individuals carrying *UBAP2L* disruptive variants (*n* = 12); ADHD, attention-deficit hyperactivity disorder; M, male; F, female; +, present; −, absent; /, no data or undetermined. (**E**) Facial features and hand abnormalities of individuals with *UBAP2L* disruptive variants. Photo credit: Shujie Zhang, Child Health Hospital of Guangxi Zhuang Autonomous Region; Chiara Leoni, Maternal Fondazione Policlinico Universitario A. Gemelli-IRCCS; Nicola Brunetti-Pierri, Telethon Institute of Genetics and Medicine (TIGEM); Claartje Meddens, University of Amsterdam; and Meredith Fuchs, Pediatrics and Genetics, Alpharetta.

*UBAP2L* is a highly constrained gene [Probability of Loss-of-function Intolerance (pLI) = 1, Loss-of-function Observed/Expected Upper bound Fraction (LOEUF) = 0] that encodes an essential regulator for SG formation (*19*, *20*). We assembled detailed genotypic and phenotypic information for a cohort of 12 individuals with *UBAP2L* de novo LGD or putative splicing variants revealed by trio or quad whole-exome sequencing (WES) (see Materials and Methods, Fig. 1, fig. S1A, and tables S1 and S2). Six variants are nonsense, three are frameshift, and three are located in the canonical (c.590+1G>A) or putative splice sites (c.703+3dup and c.3168+3A>G) (Fig. 1, A and B). None of them were reported in the gnomAD database. By minigene assay, we revealed that the splice-site variant c.590+1G>A results in the skipping of exon 7 (p.G182Efs*78), whereas the putative splice-site variant c.3168+3A>G results in the skipping of exon 26 and leads to an in-frame deletion of 66 amino acids (p.Q991_Q1056del), and the putative splice-site variant c.703+3dup results in the skipping of exon 8 (p.T198Cfs*12) (Fig. 1C). According to the exon junction complex–dependent model and start-proximal nonsense-mediated mRNA decay (NMD) insensitivity (*21*), eight nonsense/frameshift variants in *UBAP2L* identified in NDD were subject to NMD (fig. S1B). No other pathogenic variants were identified besides the *UBAP2L* variants in all families.

All affected individuals presented with neurodevelopmental concerns, such as speech-language problems, ID, childhood motor delay/hypotonia, and various behavioral issues (Fig. 1D and table S2). Specifically, 11 individuals who completed a language assessment presented with speech-language delay. Eight individuals showed borderline to severe ID among 10 individuals with intellectual evaluation, while the remaining 2 individuals did not receive a formal intellectual assessment. Eight patients exhibited various degrees of childhood motor delay. Seven of 12 individuals experienced seizures, although only 3 of 10 individuals had a formal diagnosis of epilepsy. Of the 10 patients assessed for ASD, 4 met criteria for a formal ASD diagnosis. In addition to ASD, we also observed other behavioral issues, including attention-deficit hyperactivity disorder (ADHD) (5 of 11), repetitive behavior (4 of 10), anxiety (3 of 9), and aggressive behavior (3 of 10).

We observed some facial dysmorphic features with familial variability (Fig. 1, D and E), such as abnormal palpebral fissure (four patients), deep and prominent concha (four patients), high broad forehead (three patients), hypertelorism (three patients), thin upper lip (two patients), and mild synophrys (two patients). Specifically, patient 1 shows a round face, mild synophrys, strabismus, lower-set and posteriorly rotated cupped ears, low front hairline, retromicrognathia, short neck, fetal peds, brachydactyly, and fifth finger clinodactyly at the age of 3 years and 9 months. Patient 4 shows mild synophrys, upslanting palpebral fissures, lower-set and cupped ears, mild retrognathia, fetal peds, symphalangism of thumbs, and brachydactyly at the age of 7 years. Patient 5 shows mild synophrys and micrognathia at the age of 12 years and 10 months. Patient 9 shows a triangular face, protruding ears and retromicrognathia, small palpebral fissure, and cow’s lick on the forehead at the age of 7 years and 10 months. Patient 10 shows a flat face, deep-set eyes, hypertelorism, bulbous nose, long philtrum, thin upper lip, low-set and posteriorly rotated ears, deep and prominent concha, broad forehead, nail hypoplasia, pointing finger joint contracture, and broad halluces at the age of 4 years and 2 months (Fig. 1E). In addition, patients also presented with visual impairment (6 of 10), hypotonia (4 of 8), and short stature (4 of 10). Although mildly affected, skeletal anomalies (4 of 10) and hand/foot abnormalities (5 of 10) were frequently observed, such as joint stiffness, clinodactyly of the fifth fingers, scoliosis/kyphosis, brachydactyly, nail hypoplasia, pointing finger joint contracture, broad halluces, metatarsal adducts, femoral anteversion, tibia torsion, and strephenopodia (Fig. 1, D and E, and table S2).

### NDD-related *UBAP2L* variants disrupt SG assembly

**Fig. 2.**
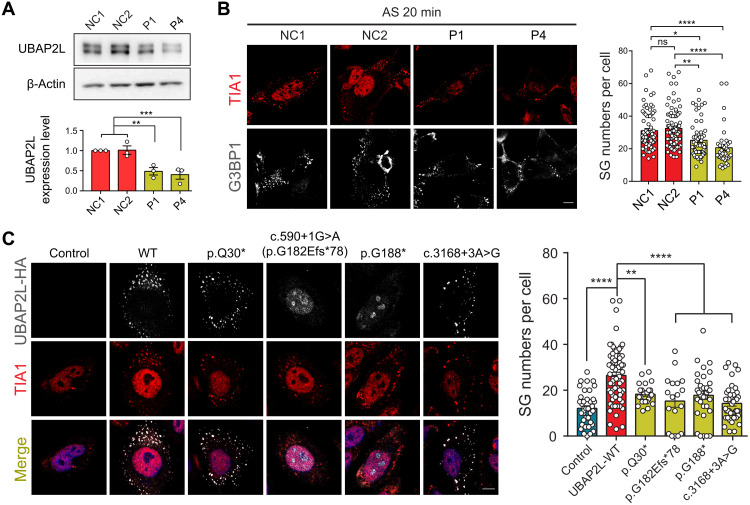
Disruptive variants in *UBAP2L* interfere with SG formation. (**A**) Immunoblotting of UBAP2L in patient-derived fibroblasts. UBAP2L proteins extracted from skin fibroblasts of healthy controls (NC1 and NC2) and patients with NDD (P1 and P4) were subjected to SDS-PAGE and immunoblotted with UBAP2L antibody. β-Actin was used as loading controls. P1 represents patient 1. P4 represents patient 4. Quantification of UBAP2L was obtained with densitometric analysis and normalized with β-actin. ***P < 0.001 and **P < 0.01; ns, not significant. (**B**) Immunofluorescence images of SGs in normal control (NC) and patient-derived fibroblasts under stress condition. Quantification and statistics of SG number per cell are shown on the right (NC1, 79 fibroblasts; NC2, 83 fibroblasts; P1, 61 fibroblasts; P4, 38 fibroblasts). (**C**) Immunofluorescence images of UBAP2L WT and variants (HA, gray) and endogenous TIA1 (red) as SG marker in transfected *UBAP2L*-KO cells under stress conditions. Quantification and statistics of SG number per cell are shown on the right (Control, 40 cells; UBAP2L-WT, 72 cells; p.Q30*, 24 cells; p.G182Efs*78, 18 cells; p.G188*, 34 cells; c.3168+3A>G, 43 cells). Scale bars, 10 μm. *P < 0.05, **P < 0.01, ***P < 0.001, and ****P < 0.0001.

As a core SG gene, *UBAP2L* regulates SG formation under several stress conditions, such as oxidative stress. We obtained the skin fibroblast cells from two affected individuals who harbor variant p.Q30* (patient 1) and variant c.590+1G>A (patient 4). We found that both variants lead to a decrease in UBAP2L protein expression (Fig. 2A). Immunofluorescence staining revealed a significant reduction of foci that were identified as SGs via labeling with well-established SG markers TIA1 and G3BP1 in fibroblast cells derived from patients compared with those in fibroblast cells from healthy individuals under the same arsenite (an oxidative stress inducer) stress (AS) (Fig. 2B). Notably, using a recombinant plasmid with a hemagglutinin (HA)–tag in the N terminus and a Flag-tag in the C terminus for variant p.Q30*, we found that the variant results in a protein that could be detected by the Flag antibody but not the HA antibody (fig. S2, A and C). We then performed protein N-terminal sequencing and confirmed that a new protein is produced lacking the normal N terminus (fig. S2B), indicating that the translation was initiated from a new ATG start codon that is located at the 11th codon downstream.

To further validate the functional effects of NDD-related variants of *UBAP2L* in SG formation, we generated *UBAP2L* knockout (KO) HeLa cell lines. Consistent with previous studies (*18*, *20*), we observed a significant reduction of SG numbers in *UBAP2L* KO cells (fig. S3). We found that KO cells transfected with mutants—including p.Q30*, c.590+1G>A: p.G182Efs*78, p.G188*, and c.3168+3A>G: p.Q991_Q1056del—show significantly fewer SGs under AS compared with cells transfected with wild type (WT) (Fig. 2C). Previous studies had demonstrated that the UBAP2L protein’s domain of unknown function (DUF) mediates the interactions between UBAP2L and G3BP1. Deletion of the DUF causes UBAP2L shuttling from the cytoplasm to the nucleus (*22*). Consistently, we found that the mutants c.590+1G>A: p.G182Efs*78 and p.G188*, leading to deletion of the DUF, presented with nuclear localization as well (Fig. 2C). These findings support that NDD-related *UBAP2L* variants have a loss-of-function consequence in SG formation and suggest SG pathology in *UBAP2L*-related NDD.

### *Ubap2l* haploinsufficiency in mouse leads to behavioral issues and cognitive impairments

**Fig. 3.**
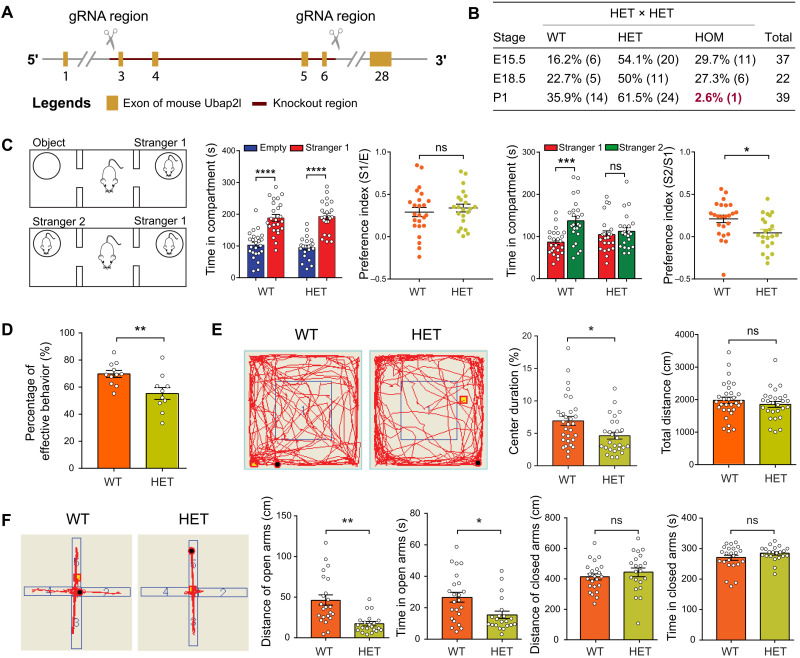
Haploinsufficiency of *Ubap2l* in mouse leads to behavioral and cognitive impairments. (**A**) Targeting strategy of *Ubap2l* conventional KO mice. (**B**) The percentage of mice with different genotypes [WT, heterozygous (HET), and homozygous (HOM)] at E15.5, E18.5, and P1. (**C**) The three-chamber test for the WT and HET mice. The time spent with empty (E), stranger 1 (S1), and stranger 2 (S2) of the WT and HET mice was recorded and compared. The preference indexes [(S1 − E)/(S1 + E) and (S2 − S1)/(S1 + S2)] were calculated and compared (*n* = 24 for WT and *n* = 22 for HET). (**D**) The Y-maze test for the WT and HET mice. The percentages of spontaneous alternation behavior of the WT and HET mice were calculated and compared (*n* = 11 for WT and *n* = 10 for HET). (**E**) The open-field test for the WT and HET mice. Time spent in the center zone and traveled distance of the WT and HET mice were recorded and compared, respectively (*n* = 31 for WT and *n* = 27 for HET). (**F**) The elevated-plus maze test for the WT and HET mice. Time spent and distance traveled in the open arms and closed arms of the WT and HET mice were recorded and compared, respectively (*n* = 23 for WT and *n* = 21 for HET). *P < 0.05 and **P < 0.01.

To investigate whether *UBAP2L* loss of function in vivo mimics NDD-related behaviors as we observed in the patients with *UBAP2L* variants, we generated a *Ubap2l* KO mouse model (see Fig. 3A and Materials and Methods). Those with homozygous deletion of *Ubap2l* (KO) presented with a lethal phenotype in a majority of embryos. Only 2.6% of KO mice survived and were distinctly undersized (Fig. 3B and fig. S4, A and B). In contrast, the heterozygous mice were viable with no morphological abnormalities.

We next conducted a battery of behavioral tests (including a three-chamber social test, open-field test, elevated-plus maze test, Y-maze test, and marble-burying test) for the heterozygous mice to assess behavioral issues and cognitive functions. WT and heterozygous mice were subjected to tests of the voluntary initiation of social interaction and the ability to discriminate social novelty (Fig. 3C). In the social interaction test, heterozygous mice showed normal social interaction and normal social preference index, indicating unaffected social interaction. In the social novelty test, in contrast, heterozygous mice spent significantly less time during interactions with the new stranger mouse and had a significantly lower social preference index compared with WT mice (Fig. 3C), indicating impaired social novelty ability. The Y-maze tests show that heterozygous mice had a decreased percent of effective alternation compared with that of WT mice, suggesting abnormal spatial working memory (Fig. 3D). The open-field and elevated-plus maze tests showed indistinguishable locomotion between WT and heterozygous mice. However, heterozygous mice spent less time in the center zone of the open-field tests and the open arm of the elevated-plus maze tests, suggesting an increased anxiolytic-like behavior (Fig. 3, E and F). In addition, we observed that heterozygous mice have mild repetitive behaviors in digging (fig. S5B). No significant differences were observed in self-grooming, marble-burying, and light-dark transition tests (fig. S5, A to D). The above results indicated that haploinsufficiency of *Ubap2l* in mice leads to low preference for social novelty, cognitive impairments, and anxious-like behaviors.

### *Ubap2l* deficiency leads to abnormal neocortex lamination and reduction of neuronal progenitor proliferation

**Fig. 4.**
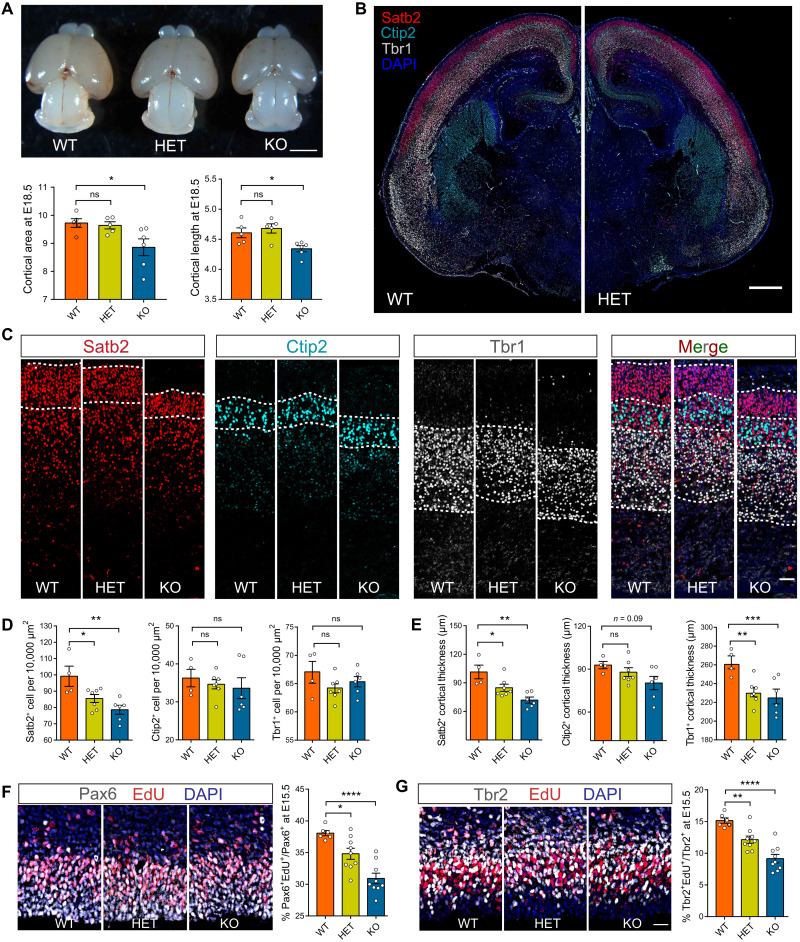
*Ubap2l* deficiency leads to abnormal cortex lamination and neural progenitor proliferation in a mouse developing brain. (**A**) Comparison of brain size between the WT, HET, and KO mice at E18.5. Cortical area and cortical length were decreased in the *Ubap2l* KO mice compared with the WT mice (*n* = 5 for WT, *n* = 5 for HET, and *n* = 6 for KO). Scale bar, 2 mm. (**B**) Immunofluorescence imaging for the coronal section of the entire cortex stained with Satb2 (upper-layer marker), Ctip2, and Tbr1 (deeper-layer marker) at E18.5. Scale bar, 0.5 mm. (**C**) The higher magnification of cortical lamination stained shown in (B). Scale bar, 50 μm. (**D** and **E**) Quantification of cells expressing Satb2 (red), Ctip2 (cyan), and Tbr1 (gray) per 10,000 μm^2^ and the length of layers expressing these markers in each section (*n* = 4 for WT, *n* = 7 for HET, and *n* = 6 for KO). (**F** and **G**) Immunofluorescence imaging of neuronal progenitors at E15.5. The brains were harvested 30 min after EdU injection and immunolabeled with EdU, radial progenitors (Pax6), and intermediate progenitors (Tbr2). Percentage of cells expressing Pax6 and Tbr2 colabeled with EdU was calculated and compared (*n* = 6 for WT, *n* = 9 for HET, and *n* = 9 for KO). Scale bar, 25 μm. *P < 0.05, **P < 0.01, ***P < 0.001, and ****P < 0.0001.

UBAP2L regulates SG dynamics, which are important for gene expression regulation. Growing evidence indicates that disturbed gene regulation interferes with neocortical neurogenesis, which contributes substantially to NDD pathogenesis. To investigate the role of *Ubap2l* in embryonic cortical neurogenesis, we first evaluated the overall brain size and cortical cytoarchitecture. We determined that KO embryos are viable at around embryonic day 18.5 (E18.5) but show a significant decrease in cortical length and cortical area compared with littermate WT mice (Fig. 4A). No significant difference was observed in the heterozygous embryos.

We then investigated whether deletion of *Ubap2l* affected neocortex neurogenesis. Lamina-specific markers were used to calculate neuron density and layer thickness in the cortices of WT, heterozygous, and KO embryonic cortex at E18.5. We observed decreased thickness of the deeper-layer cortex labeled by deeper-layer–specific markers Tbr1^+^ and Ctip2^+^ in both the heterozygous and KO cortex. No changes were observed in the density of Tbr1^+^ and Ctip2^+^ cells. We then performed immunohistochemistry of layer-specific marker Satb2 in the neocortex at E18.5. We observed that both the thickness and the number of Satb2^+^ cells were significantly reduced (Fig. 4, B to E, and fig. S4C). Together, these data demonstrate that loss of *Ubap2l* leads to disordered cortical development and lamination.

In the developing cortex, the deeper- and upper-layer neurons were generated through self-renewal and transit amplification of neuronal progenitors (*23*). We next assessed the number of proliferating progenitors at E15.5, the peak period of neurogenesis in mouse. Proliferating radial glia cells in the S phase were pulse-labeled with 5-ethynyl-2′-deoxyuridine (EdU) and fluorescent-labeled with anti-Pax6 antibody. We revealed a significant decrease in the number of EdU^+^Pax6^+^/Pax6^+^ cells in both the heterozygous and KO cortices (Fig. 4F). During neurogenesis, radial glia cells divide asymmetrically to generate the intermediate progenitor cells, which contribute to the expansion of the neocortex. Intermediate progenitor cells can be molecularly distinguished from radial glia cells by their expression of Tbr2 (*24*). We then assessed the effect of *Ubap2l* deficiency in the proliferation of intermediate progenitor cells. We observed reduced proliferation of Tbr2^+^ cells in both the heterozygous and KO cortices (Fig. 4G). These data suggest that loss of *Ubap2l* leads to perturbation of radial glia cells and intermediate progenitor cell proliferation.

### Dysregulation of SG dynamics during neurogenesis in *Ubap2l*-deficient mice

**Fig. 5.**
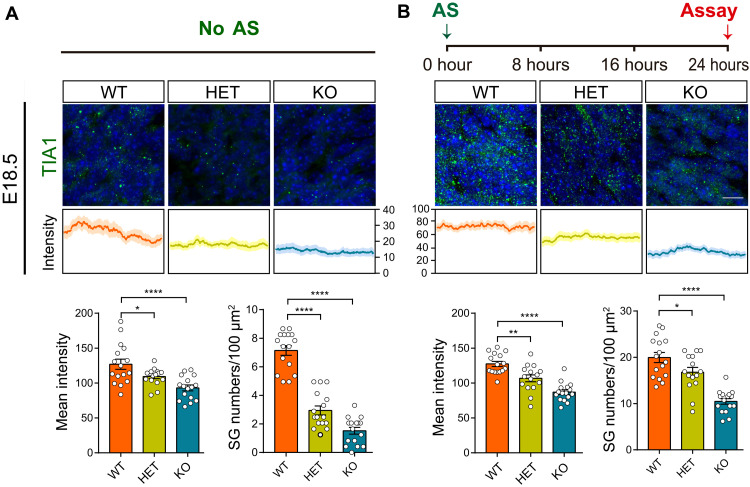
Disruption of SG formation in *Ubap2l*-deficient mouse brain. (**A**) Immunofluorescence imaging of the coronal section of the WT, HET, and KO mice brain labeled with the SG marker TIA1 at E18.5 without stress condition. Newborn neurons in the cortical plate were assayed. Quantification of the mean intensity of TIA1 immunosignals and SG numbers per 100 μm^2^ is shown, respectively (*n* = 16 for WT, *n* = 16 for HET, and *n* = 16 for KO). (**B**) Immunofluorescence imaging of the coronal section of the WT, HET, and KO mice brain labeled with the SG marker TIA1 at E18.5 under stress conditions. Newborn neurons in the cortical plate were assayed. Quantification of the mean intensity of TIA1 immunosignals and SG numbers per 100 μm^2^ is shown, respectively (*n* = 16 for WT, *n* = 15 for HET, and *n* = 16 for KO). Scale bar, 10 μm. *P < 0.05, **P < 0.01, and ****P < 0.0001.

SGs exist in neurons and embryos where there are substantial pools of untranslating messenger ribonucleoprotein (*25*). However, whether SG dynamics are impaired during early brain development has not been well characterized. To investigate whether *Ubap2l* deficiency in mouse leads to disturbed SG dynamics during early neurodevelopment, we euthanized the *Ubap2l*-deficient embryos under the normal physiological environment at E18.5. We observed considerable TIA1-positive SGs in the developing cortex, indicating that there are stress events under normal physiological conditions. Notably, we observed decreased SG intensity and numbers in both the heterozygous and KO cortices compared with WT cortex (Fig. 5A). To further validate that SG formation is disturbed in the *Ubap2l*-deficient mouse, we injected AS (4 mg/kg) into pregnant mice and euthanized the embryos 24 hours thereafter. We observed significantly increased SG numbers under AS. Again, the intensity and number of SGs were decreased in both the heterozygous and KO cortices when compared with WT cortices (Fig. 5B). These in vivo data suggest that *Ubap2l* deficiency impaired SG assembly during cortical development, and the findings further support the SG pathology in neurodevelopment and NDDs.

### Significant enrichments of SG genes in NDD gene sets

The above data warranted us to further explore whether SG disturbance is a common pathology involved in NDDs. We first assessed the enrichment of SG-related genes in NDD genes. We defined the SG gene sets based on the findings from three representative publications that detected SG regulatory and constituent components using proteomics and high-throughput genome-wide screening approaches (see Materials and Methods and fig. S6) (*13*, *17*, *18*). The total set is the combination of the two SG component lists and one SG regulator list (total set) (*n* = 843; table S3), and the core set is the shared genes among the three gene lists (*n* = 26; table S3 and fig. S6).

We introduced two well-established NDD gene lists. One is the well-curated high-confidence ASD gene list (361 SFARI genes within gene score of S or 1) from the SFARI (Simons Foundation Autism Research Initiative) Gene database (https://gene.sfari.org/). The other one is a well-curated developmental disorder gene list (827 genes with “definitive” or “strong” evidence) from the DDG2P database (www.deciphergenomics.org/ddd/ddgenes) (see Materials and Methods and table S4). We uncovered a significant enrichment of SG genes (total set) [SFARI genes: odds ratio (OR) = 2.33, *P* = 4.762 × 10^−5^; DDG2P genes: OR = 2.13, *P* = 3.548 × 10^−7^] and SG assembly genes (SFARI genes: OR = 2.19, *P* = 0.0305; DDG2P genes: OR = 2.27, *P* = 0.0010) in both NDD gene sets (table S5). The enrichment signal became stronger when we performed the analysis for the SG core set genes (SFARI genes: OR = 13.11, *P* = 9.219 × 10^−5^; DDG2P genes: OR = 3.03, *P* = 0.0947). In addition, we also observed significant enrichments of SG gene sets in the binding targets of well-characterized NDD-associated RNA binding proteins such as FMRP, RBFOXs, and CSDE1 (*26*–*29*) or DNA binding protein such as CHD8 (see Materials and Methods and table S5) (*30*, *31*).

### Newly implicated SG-related NDD genes and network enrichment

To further identify specific SG-related NDD genes, we leveraged DNMs from 40,853 NDD probands, including 9228 individuals primarily diagnosed with ASD and 31,625 individuals primarily diagnosed with ID or DD from 26 published studies (see Materials and Methods and table S6). In total, 3410 variants in the coding regions of 843 SG genes were annotated (table S7) and included in the following analysis.

**Fig. 6.**
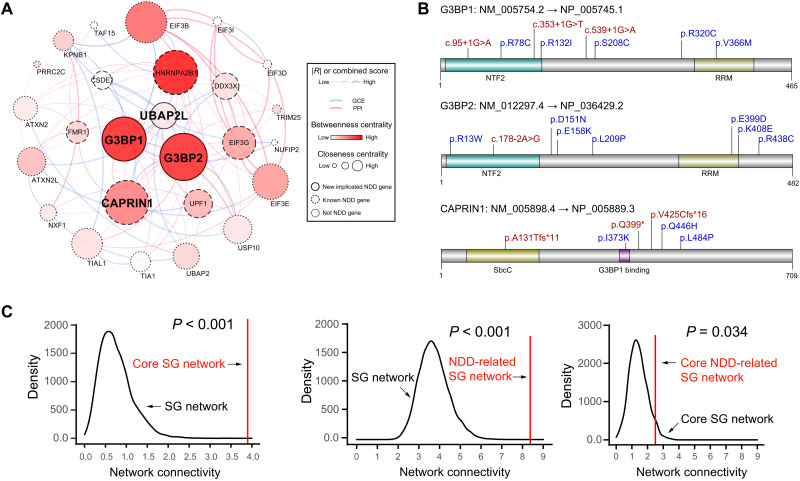
Newly implicated core SG–related NDD genes and network enrichment analysis. (**A**) The PPI and GCE network of 26 core SG genes. The color and size of each gene node represent the closeness and betweenness centrality in the network, respectively. The color of the edge line represents the type of network (GCE: blue; PPI: red). The width of the edge line represents the strength of interaction (|*R*| for GCE and combined score for PPI). Borderline types of the gene nodes represent different gene sets. (**B**) Schematic representation of de novo LGD (red) and missense (blue) variants in three SG-essential genes: *G3BP1*, *G3BP2*, and *CAPRIN1*. (**C**) Downsampling analysis of network edge density between networks formed by different gene sets. *P* values were calculated by empirically estimating the probability of observing a denser network by randomly sampling 10,000 subnetworks of the same counts of genes from background interactome.

We introduced two statistical models, DenovolyzeR (*32*) and the chimpanzee-human divergence model (CH model) (*33*), to perform enrichment analysis for de novo LGD, missense, and protein-altering (LGD + missense) variants in the NDD cohort in comparison with the expected numbers of DNMs in the general population. Using the most stringent exome-wide multiple testing correction (Bonferroni correction for ~19,000 genes), we observed significant excess (corrected *P* < 0.05) of DNM in 31 SG genes (table S8). Among them, 24 genes are known NDD genes. Seven genes (*ETF1*, *PCBP2*, *QKI*, *RPS5*, *RPL13A*, *VEGFB*, and *SRSF1*) were not implicated in previous studies, and none of them is in the core SG gene set. To prioritize new candidate genes for follow-up, we introduced the false discovery rate (FDR) approach to perform the multiple testing correction for the 843 SG genes we analyzed in this study. This strategy prioritizes 72 genes with FDR less than 0.05 (FDR correction for 843 SG genes) (Table 1, fig. S7A, and table S8). Among them, 43 genes have been implicated in NDD in previous studies, and 29 genes are newly implicated (Table 1), including the 3 core SG genes *G3BP1*, *G3BP2*, and *UBAP2L* (Table 1 and Fig. 6, A and B). As expected, the newly implicated or known NDD-related SG genes are more intolerant in terms of gnomAD pLI (new: *P* = 0.025, known: *P* = 1.17 × 10^−5^) and missense *Z* score (new: *P* = 0.024, known: *P* = 2.1 × 10^−11^) (fig. S7B) compared with SG genes not implicated in NDDs.

**Table 1.** SG-related gene showing nominally significant (FDR < 0.05) excess of DNM in 40,853 NDD families. Newly implicated genes are in bold. Newly implicated genes with significance in both models are in bold and underlined. “LGD and MIS” represents significance that is observed in both LGD and missense variants. “LGD only” represents significance that is observed in LGD variants but not in missense variants. “MIS only” represents significance that is observed in missense variants but not in LGD variants. “(LGD + MIS) only” represents significance that is only observed when considering LGD variants and missense variants together.

**Category**	**FDR ≤ 0.01**	**0.01 < FDR < 0.05**
LGD and MIS	*CAPRIN1 DDX3X EP300 HNRNPU NONO* ***PCBP2*** *PURA RPS6KA3* ***SRSF1*** *TLK2*	*ANKRD17*
LGD only	*CELF2 CNOT1 GIGYF1 HNRNPD HNRNPK* ***ILF2*** *LARP4B MSL2* ***NCL*** *SMAD6 SYNCRIP TNRC6B* ***UBAP2L***	*CSDE1 CTNND1 EIF4A2* ***G3BP1*** *HNRNPA2B1 PQBP1 TRIM8 UPF3B*
MIS only	*AGO1 CALM1* ***CSE1L*** *DDX6 DYNC1H1 EEF2* ***EIF4G2*** *EIF5A* ***ETF1*** ***G3BP2*** *GNB2 MTOR PPP2R1A PRPF18* ***QKI*** ***RPL13A*** ***RPS5*** *TNPO2* ***TPM2*** *TRPM3* ***VEGFB***	*CACNA1G CTNNA2* ***HIST1H4J*** ***MAP4*** *RPL10* *U2AF2* *UPF1* ***VCP***
(LGD + MIS) only	***MSI1***	***CPSF7*** ***DAZAP1 EPPK1 HSP90AA1 IK LRPAP1 MTIF2 PUM2 SF1 YES1***

The 26 core SG genes form a highly connected protein-protein interaction (PPI) and gene coexpression (GCE) network (Fig. 6A). The network edge density is significantly higher among NDD-related SG genes when compared to all SG genes (Fig. 6C). Under the background network of core SG genes, the network was formed by NDD-related core SG genes, including three newly implicated NDD genes (*G3BP1*, *G3BP2*, and *UBAP2L*) and seven known NDD genes (*CAPRIN1*, *CSDE1*, *DDX3X*, *EIF3G*, *FMR1*, *UPF1*, and *HNRNPA2B1*) (Fig. 6A), which show significantly higher network edge density (*P* = 0.034) (Fig. 6C). These data further support the association of NDD genes in the SG network.

### DNMs in SG essential genes interfere with SG assembly and disrupt the network interaction

*G3BP1* and *G3BP2* have the highest centrality within the core SG network (Fig. 6A) and play crucial roles in SG assembly (*34*). There are three de novo splice-site variants (c.95+1G>A, c.353+1G>T, and c.539+1G>A) and five de novo missense variants (p.R78C, p.R132I, p.S208C, p.R320C, and p.V366M) identified in *G3BP1* (Fig. 6B). We observed an excess of de novo LGD (*P* = 0.0002, FDR = 0.013) and missense (*P* = 0.0016, FDR = 0.058) variants of *G3BP1* in the CH model but not in DenovolyzeR (LGD: *P* = 0.004, FDR = 0.22; missense: *P* = 0.005, FDR = 0.23) (table S8). However, for protein-altering variants (LGD + missense), excessive burdens are observed in both models (CH model: *P* = 6.44 × 10^−6^, FDR = 0.00034; DenovolyzeR: *P* = 0.0001, FDR = 0.0064). There is one de novo splice-site variant (c.178-2A>G) and seven de novo missense variants (p.R13W, p.D151N, p.E158K, p.L209P, p.E399D, p.K408E, and p.R438C) in *G3BP*2 (Fig. 6B), which drove a nominally significant enrichment of de novo missense variants in both models (CH model: *P* = 2.5 × 10^−5^, FDR = 0.0018; DenovolyzeR: *P* = 0.00025, FDR = 0.024) (table S8).

**Fig. 7.**
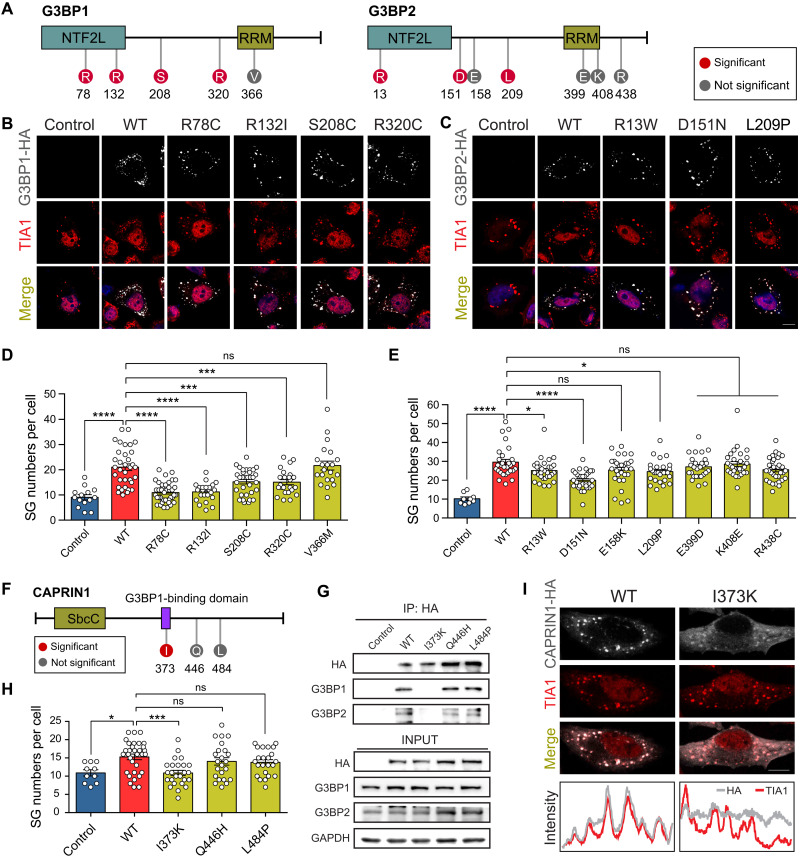
De novo missense variants in *G3BP1*, *G3BP2*, and *CAPRIN1* disrupt SG formation and network interaction. (**A**) Schematic protein structure of G3BP1 and G3BP2 showing the NTF2L domain and RRM region. The positions of de novo missense variants affecting SG formation are shown in red. The positions of de novo missense variants unaffecting SG assembly are shown in gray. (**B** and **C**) Immunofluorescence imaging of the WT and mutant proteins of G3BP1/2 (HA, gray) and endogenous TIA1 (red) as SG markers in transfected *G3BP1/2* KO cells under stress conditions. Scale bar, 10 μm. (**D** and **E**) Quantification of SG numbers per cell for G3BP1/2 WT and variants. (D) Control, 14 cells; WT, 34 cells; R78C, 41 cells; R132I, 23 cells; S208C, 33 cells; R320C, 24 cells; V366M, 21 cells. (E) Control, 9 cells; WT, 30 cells; R13W, 32 cells; D151N, 32 cells; E158K, 29 cells; L209P, 28 cells; E399D, 27 cells; K408E, 30 cells; R438C, 32 cells. (**F**) Schematic protein structure of CAPRIN1 showing the domain of SbcC and G3BP1 binding. The positions of de novo missense variants that affected (red) and did not affect (gray) SG assembly are shown. (**G**) Coimmunoprecipitation assay of CAPRIN1 mutant proteins and G3BP1/2. CAPRIN1-HA-IP lysate from *CAPRIN1* KO HeLa cells transfected with WT and mutant constructs was subjected to SDS-PAGE and immunoblot with G3BP1/2. Input was applied as protein-level controls. (**H**) Quantification of SG numbers per cell for CAPRIN1 WT and variants (Control, 10 cells; WT, 32 cells; I373K, 25 cells; Q446H, 23 cells; L484P, 23cells). (**I**) Immunofluorescence imaging of WT and mutant proteins of CAPRIN1 (HA, gray) and endogenous TIA1 (red) as SG marker in transfected *CAPRIN1* KO cells under stress conditions. Scale bar, 10 μm. Immunofluorescence intensity lines of CAPRIN1 WT, CAPRIN1 I373K mutant, and SG marker TIA1 are plotted. *P < 0.05, ***P < 0.001, and ****P < 0.0001.

To replicate the function of *G3BP1* and *G3BP2* in SG formation, we generated *G3BP1* or *G3BP2* KO HeLa cell lines (fig. S8A). We performed immunofluorescence analysis with transfected KO cells under the AS condition. Consistent with previous studies (*18*), we observed a significant reduction of SG numbers in both *G3BP1* and *G3BP2* KO cells (fig. S8, B and C). To assess the impact of NDD-related variants in *G3BP1* and *G3BP2* on SG formation, we calculated the SG numbers in HeLa cells expressing C-terminal HA-tagged G3BP1/2 WTs, five G3BP1 mutants (R78C, R132I, S208C, R320C, and V366M), and seven G3BP2 mutants (p.R13W, p.D151N, p.E158K, p.L209P, p.E399D, p.K408E, and p.R438C) (Fig. 7A). HA-tagged G3BP1 and G3BP2 mutant proteins show identical subcellular distribution to endogenous G3BP1/2 proteins under AS (Fig. 7, B and C). However, we found that four in five *G3BP1* variants (p.R78C, p.R132I, p.S208C, and p.R320C) and three in seven *G3BP2* variants (p.R13W, p.D151N, and p.L209P) resulted in significantly fewer SG formations under AS compared with WT (Fig. 7, B to E). These findings suggest that NDD-related variants in *G3BP1* and *G3BP2* disturb SG formation.

In the core SG gene network, *CAPRIN1* is located in the central position and has tight connection with other SG hub genes, such as *G3BP1* and *G3BP2* (Fig. 6A). Although the genetic evidence is still not very convincing, *CAPRIN1* had been implicated as an NDD risk gene (*3*, *35*). We observed a significant excess of DNMs in *CAPRIN1* by the CH model (LGD: *P* = 1.611 × 10^−14^, FDR = 3.02 × 10^−12^; missense: *P* = 4.03 × 10^−12^, FDR = 5.23 × 10^−10^) but not the DenovolyzeR model (table S8). Three de novo missense variants, including p.I373K, p.Q446H, and p.L484P, were identified in *CAPRIN1* (Fig. 7F). Notably, variant p.I373K is located in the G3BP1-interacting domain. It is well established that CAPRIN1 binding to G3BP1/2 promotes SG formation (*36*). To test whether *CAPRIN1* de novo missense variants disrupt the interaction between CAPRIN1 and G3BP1/2 and interfere with SG formation, we generated *CAPRIN1* KO HeLa cell lines (fig. S8A). We observed a significant reduction of SG numbers in KO cell lines, which is in line with previous studies (fig. S8, Band C) (*18*). Notably, coimmunoprecipitation revealed a complete abolishment of interaction between CAPRIN1 p.I373K mutant proteins and G3BP1/2 (Fig. 7G). Unlike WT proteins that localized in SGs via colabeling with TIA1, p.I373K mutant proteins are diffusely distributed in the cytoplasm under AS (Fig. 7I). We consistently observed decreased SG formation in cells with p.I373K variant (Fig. 7, H and I). These data indicate that NDD-related variants disrupted the interaction of the core SG network and further support the hypothesis that SG disturbance is a common pathology in NDDs.

## DISCUSSION

The human brain is the most complex yet highly ordered structure. Gene expression homeostasis is essential for embryonic brain development. A substantial amount of physiological and external stress conditions occur during brain development. Research with a variety of animal models has shown that stress conditions, such as oxidative stress, play a critical role in controlling radial progenitor cell proliferation and differentiation, cortical neurogenesis, and neuronal organization (*37*). The study of human organoids also revealed that oxygen deprivation on corticogenesis affects intermediate progenitor proliferation and underlies long-term neurodevelopmental impairment (*38*).

SGs are membraneless compartments in eukaryotic cells that are dynamically induced upon varieties of stress conditions (*12*) and thought to be critical to maintain gene expression homeostasis during embryonic brain development (*25*, *39*). UBAP2L is an essential regulator for SG assembly (*17*, *19*). During SG formation, UBAP2L is recruited to immature SGs and acts upstream of G3BP1 and facilitates G3BP1 core formation (*19*, *20*). Direct interactions between the UBAP2L DUF domain and G3BP1 are essential for SG formation (*22*). Loss of UBAP2L reduces the number of SG foci as observed in previous studies (*19*, *20*) and this study. We validated that the NDD-related LGD variants are loss-of-function variants in terms of SG formation, which indicates the SG pathology in NDD risk. The 12 patients with *UBAP2L* variants manifest a consistent phenotypic profile, including language/motor delay and ID, although the severity and neurodevelopmental phenotype spectrum are variable and broad, which was also observed in other NDD genes regulating SG assembly, such as *CSDE1* (*26*) and *FMR1* (*40*). Given that SG formation alters translation, the deficit of SG assembly genes may lead to similar phenotypes with genes whose function is influenced by translation rates, such as *UPF3B* and *UPF2*. *UPF3B*- and *UPF2*-related disorders recapitulate many of the phenotypes observed in patients carrying the *UBAP2L* variant, including mild to severe ID, autism-like behaviors, and seizures (*41*, *42*). Our *Ubap2l* haploinsufficiency mouse model shows moderate DD and behavioral defects, including social deficits and cognitive impairments, which mimic the phenotypes observed in patients. The homozygous mice are more severely affected, indicating the critical roles of *Ubap2l* in development. Together, these lines of evidence strongly support the notion that haploinsufficiency of *UBAP2L* leads to a new NDD.

We provided both in vitro and in vivo evidence that *UBAP2L* loss-of-function events lead to a significant decrease in SG formation, which presumably leads to neurogenesis disruption. *UBAP2L* loss-of-function variants lead to significantly reduced SG formation in the patient-derived cell lines under stress conditions. Both *Ubap2l* heterozygous and KO mice manifest remarkable reduced SG formation accompanied with abnormal cortical lamination and progenitor proliferation. This is consistent with the phenotype observed in stress-induced human three-dimensional cellular models and mammalian cortex of dysregulation of oxidative stress–responsive genes (*37*, *38*).

The SG disturbance in cell lines with *UBAP2L* LGD variants, Ubap2l haploinsufficiency, and KO mice suggests the SG defect underlying the pathogenesis of NDD. The high frequency of stress conditions in early brain development and the association of possible stress-related environmental factors with NDD risk inspired us to propose the hypothesis that variants in SG genes associate with NDD risk by disturbing SG dynamics in early brain development and are a common pathway leading to NDD. Combining DNMs of SG genes from more than 40,000 NDD families, we reported an excess of DNMs in 72 SG genes. Among them, 29 genes including *UBAP2L* had not been implicated as NDD candidate genes in previous studies. Notably, two other essential genes for SG formation, *G3BP1* and *G3BP2*, also reach a nominally significant threshold in both statistical models. SGs are formed to shut down gene translation in cells under stress conditions via a complex network of PPI and protein-RNA interactions (*36*). The central hub of this interacted network is *G3BP1* and its homolog *G3BP2* (*18*).

In addition to the association in terms of the human genetics, the functional assays also support the association of *G3BP1/2* variants with NDDs. Of the seven de novo missense variants in *G3BP1/2* affecting SG formation, all three missense variants located in the NTFL domains (two from G3BP1 and one from G3BP2) lead to reduced SG numbers. Previous studies showed that the NTF2L domain plays a critical role in dimerization and interaction with other SG core components, such as CAPRIN1 and UBAP2L, and G3BP1 lacking the NTF2L domain failed to rescue SG assembly in G3BP1/2 double-KO cells (*43*). Thus, it is possible that the variants in this domain disturb the interaction between G3BP1/2 and its interacting components during SG formation, such as CAPRIN1 and UBAP2L. G3BP1/2 contain a central segment predicted to be intrinsically disordered regions (IDRs). In addition to variants in the NTF2L domain, four missense variants in IDR were also found to be associated with reduced SG numbers. IDRs were regarded as an essential factor in SG assembly (*44*). These de novo missense variants might affect SG formation by influencing IDR-stabilized scaffold function.

Another important piece of evidence supporting SG pathology in NDDs comes from the functional effect of de novo missense variants in the G3BP-interacting domain of *CAPRIN1*. CAPRIN1 has been proven to be crucial in SG assembly, mostly by interacting with G3BP in the highly interacted network for SG formation. CAPRIN1 was identified as a prominent regulator of G3BP-mediated assembly that promotes SG formation. It could increase the RNA binding affinity of G3BP by interacting with the NTF2 domain of G3BP (*36*). The de novo missense variant (I373K) in the G3BP-interacting domain of CAPRIN1 completely abolishes this interaction and disrupts SG formation.

In addition to the core SG genes (*UBAP2L*, *G3BP1*, and *G3BP2*) for which we explored the functional effect, our analysis also implicates 26 additional, new NDD risk genes that are constituent or regulatory components and show nominal significance. Although the function of these genes in SG formation and dynamics has not been thoroughly investigated, some of them—such as *LRPAP1*, *MTIF2*, *HIST1H4J*, *RPS5*, and *VEGFB*—have been proven to regulate SG formation (*18*), and the mutation pattern of these genes in NDDs is interesting (fig. S9) as missense variants were observed to cluster in certain regions or potentially novel functional domains. Functional analysis of their roles in neurodevelopmental and SG dynamics will provide further understanding of the SG pathology in NDDs. In the current study, we did not analyze the rare inherited variants of SG genes because of the lack of data. Rare inherited variants in SG genes might also be risk factors for NDD and deserve further investigation.

Together, our data provide evidence for a strong association between SG essential genes and NDDs from both genetic and functional perspectives and offer dozens of NDD candidate genes for future investigation. Considering that embryonic exposure to harmful environmental stressors—including oxidative stress, infection, malnutrition, and maternal stress during pregnancy—has been reported to increase the risk of NDDs (*45*–*47*), our study also suggests that SGs might be important mediators to investigate gene-environment interactions conferring NDD risk.

## MATERIALS AND METHODS

### Clinical assessment and molecular genetic methods for affected families

Families with *UBAP2L* LGD variants were recruited to different collaborating institutes through a large-scale international collaboration connected by GeneMatcher (*48*) or personal communications. For each affected individual, detailed clinical information was collected by neurologists, psychiatrists, pediatricians, geneticists, or genetic counselors. Genomic DNA was extracted from the whole blood of the affected individuals and their parents. Trio-based WES was performed to identify the variants in *UBAP2L*. Detailed methods of WES and data analysis for the 12 families are described in Supplementary Notes. Written informed consent was obtained from study participants or their parents or legal guardians, in line with local institutional review board (IRB) requirements at the time of collection. The IRB of the Central South University approved this study (#2019-1-17). All procedures were in accordance with the ethical standards of the responsible committee on human experimentation (institutional and national).

### Minigene assay for putative splice-site mutations

Two genomic fragments containing exons 5 to 8 and exons 25 to 27 were amplified from normal control DNA, respectively, by PrimeSTAR HS DNA polymerase (Takara) according to the manufacturer’s protocols, with primers carrying restriction sites. These DNA inserts were cloned into the splicing vector pcDNA3.1myc-His(+)A by an EasyGeno Assembly Cloning kit (TIANGEN). The mutant plasmids were constructed by a Q5 Site Directed Mutagenesis kit (NEB) with site-specific mutant primers. WT and mutant constructs were verified by Sanger sequencing.

To avoid false-negative results, we used two kinds of cell lines, including human embryonic kidney (HEK) 293 and HeLa cells. The cell lines were cultured in Dulbecco’s modified Eagle’s medium (DMEM) containing 10% fetal bovine serum (FBS), 1% nonessential amino acids, 2 mM glutamine, and 1% penicillin/streptomycin stock solution at 37°C, 5% CO_2_, and 95% humidity. The HEK293 and HeLa cells were grown to approximately 90% confluency in six-well plates and transiently transfected with 2 mg of KO and mutant plasmids using jetPRIME Transfection Reagent (Polyplus) according to the manufacturer’s instructions. After 36 hours of culturing, total RNA was extracted using the TRIzol reagent (Invitrogen) according to the manufacturer’s protocols. Complementary DNA (cDNA) synthesis was carried out with 2 μg of RNA by the FastQuant cDNA First Strand Synthesis Kit (TIANGEN) following the standard protocol. To evaluate splicing, polymerase chain reaction (PCR) was performed using Taq DNA polymerase (Takara), with specialized primers. The PCR products were cloned into the pMD19-T vectors (Takara), and DH5α competent cells were transformed with these pMD19-T constructs. After 16 hours of culturing, we picked up these colonies and identified their transcripts by Sanger sequencing.

### Construct *G3BP1*/*G3BP2*/*CAPRIN1*/*UBAP2L* KO HeLa cell lines with CRISPR-Cas9

Guide RNA (gRNA) sequences for CRISPR-Cas9 were designed at the CRISPR design website (http://crispr.mit.edu/), provided by the Feng Zhang Lab. Insert oligonucleotides for human G3BP1 gRNA are 5′-CACCGTGTCCGTAGACTGCATCTGC-3′ and 5′-AAACGCAGATGCAGTCTACGGACAC-3′; those for human G3BP2 gRNA are 5′-CACCGTCTGGTTTAGCTTCGACTCT-3′ and 5′-AAACAGAGTCGAAGCTAAACCAGAC-3′; those for human CAPRIN1 gRNA are 5′-CACCGAGTGCCAATATTGTCCGAAG-3′ and 5′-AAACCTTCGGACAATATTGGCACTC-3′; and those for human UBAP2L gRNA #1 and #2 are 5′-CACCGCCTCAAAGTCAGCATCATTA-3′/5′-AAACTAATGATGCTGACTTTGAGGC-3′ and 5′-CACCGTAGACTTGCACAGATGATTT-3′/5′-AAACAAATCATCTGTGCAAGTCTAC-3′, respectively. The complementary oligonucleotides for gRNAs were annealed and cloned into lentiCRISPRv2 vector (Addgene). To make lentivirus, lentiCRISPRv2/gRNA #1 or lentiCRISPRv2/gRNA #2 plasmids were cotransfected into HEK293T cells with the packaging plasmids pMD2G (Addgene, 12259) and psPAX2 (Addgene, 12260). HeLa cells were infected with either lentiCRISPRv2 or gRNA lentivirus. Two days after infection, cells were treated with puromycin (2 μg/ml) to select the KO-positive cells for 4 days until the cells stopped dying. We replanted the positive cells in a 96-well plate with a seeding density of 0.6 cells per well and isolated the single clones to culture after 2 weeks. The KO HeLa cell lines were verified by Western blot. The 293T cell lines and HeLa cell lines were obtained from the National Collection of Authenticated Cell Culture of China (293T accession number: GNHu17; HeLa accession number: TCHu187).

### Cell culture, SG induction, and immunofluorescence

Skin fibroblast cells (NC1, species: *Homo sapiens*, tissue: foreskin, sex of origin: male child; NC2, species: *H. sapiens*, tissue: foreskin, sex of origin: male adult; P1, species: *H. sapiens*, tissue: thigh skin, sex of origin: male child; P4, species: *H. sapiens*, tissue: skin, sex of origin: female child), HeLa cells, and 293T cells were cultured in DMEM supplemented with 10% FBS. Cells were cultured in a 24-well plate with a coverslip in each well and transfected with plasmids using Lipofectamine 3000. SGs were induced by treatment with AS (500 μM for 30 min, unless specifically indicated in the figure legends). Cells were fixed for 10 min in 4% paraformaldehyde (PFA), then rinsed with 1× PBS (phosphate-buffered saline), and blocked with permeabilization and blocking buffer [1× PBS, 3% bovine serum albumin (BSA), 10% FBS, and 0.2% Triton]. Cells were incubated with primary antibodies overnight at 4°C. After PBS washing, the cells were incubated with fluorochrome-labeled secondary antibodies. The slides were examined after mounting with 4′,6-diamidino-2-phenylindole (DAPI) under the fluorescence microscope. Cells with SGs were randomly selected from each group. The analyses were double-blinded.

### Antibodies

The antibodies used for Western blot analysis and immunofluorescence included mouse anti-HA (1:2000 for Western blot and 1:800 for immunofluorescence; H3663) from Millipore-Sigma, mouse anti–glyceraldehyde-3-phosphate dehydrogenase (GAPDH) (1:10,000; g9295) from Millipore-Sigma, mouse anti-G3BP1 (1:500 for Western blot and 1:200 for immunofluorescence; sc-365338) from Santa Cruz Biotechnology, rabbit anti-G3BP2 (1:2000; ab86135) from Abcam, rabbit anti-TIA1 (1:300; 12133-2-AP) from Proteintech, rabbit anti-CAPRIN1 (1:2000; 15112-1-AP) from Proteintech, rabbit anti-UBAP2L (1:4000; ab70319) from Abcam, mouse anti-actin (1:10,000; A5441) from Millipore-Sigma, mouse anti-tubulin (1:1000; T8328) from Millipore-Sigma, mouse anti-Satb2 (1:100; ab51502) from Abcam, rat anti-Ctip2 (1:200; ab18465) from Abcam, rabbit anti-Tbr1 (1:200; ab31940) from Abcam, rabbit anti-Pax6 (1:200; 901301) from BioLegend, and rabbit anti-Tbr2 (1:200; ab23345) from Abcam.

### Coimmunoprecipitation and Western blot

Cells were lysed by NP-40 solution with protease inhibitor; the mixture was shaken at 4°C for 30 min. The supernatant was obtained after centrifugation at 13,000 rcf for 5 min, and 50 μl of supernatant was collected as Input group. Total supernatant was incubated with HA magnetic beads overnight at 4°C. The beads were rinsed three times with buffer PBST and eluted proteins with 4% SDS and 5× loading buffer. Then, Input and immunoprecipitation (IP) groups were subjected to Western blot. Total proteins and Input or IP group were separated by 10% SDS–polyacrylamide gel electrophoresis (PAGE) and transferred to polyvinylidene difluoride membranes. The membranes were blocked with 5% nonfat dry milk for 1 hour at room temperature and incubated with primary antibodies overnight at 4°C and secondary antibodies for 1 hour at room temperature. Last, blots were developed with enhanced chemiluminescence and quantified by densitometry in ImageJ. Coimmunoprecipitation experiment was replicated three times.

### *Ubap2l* KO mice

The *Ubap2l* conventional KO mouse line was bred on C57BL/6N background and generated by Cyagen Biosciences (serial number: KOCMP-03049-ubap2l). Animals were group-housed with food and water ad libitum under a 12-hour light/12-hour dark cycle. The genotype was determined by PCR analysis using genomic DNA extracted from the mice. WT allele had one band with 615 base pairs (bp) (forward primer sequence: 5′-ACAAAGACCACTCAGTTATGGTAG-3′; reverse primer sequence: 5′-GACACAAACAGTGGAGACAGGAAG-3′). Homozygotes had one band with 800 bp (forward primer sequence: 5′-ACAAAGACCACTCAGTTATGGTAG-3′; reverse primer sequence: 5′-AGCATTCCTTAGCATCACTCTTGG-3′). Heterozygotes had two bands with 800 and 615 bp.

### Behavioral tests

Approximately 6-week-old male mice were used for testing. All the behavior tests were performed between 2:00 p.m. and 6:00 p.m. in a quiet environment. Mice were bred in a room 1 week before the first test in order for mice to adapt to the environment. There was an interval of 2 to 3 days between two behavioral tests. Each mouse was not subjected to more than four behavioral tests. All behavioral tests were done blinded to genotypes. Experiments were performed in accordance with an animal study protocol approved by the Institutional Animal Care and Use Committee in compliance with the Association for Assessment and Accreditation of Laboratory Animals guidelines. Study protocols complied with all relevant ethical regulations and were approved by the IRB of Central South University (#2019-2-23).

#### Three-chamber social test

The three-chamber social test assessed the social interaction and social novelty ability of mice (*49*). This assay consisted of three compartments with two cages in the left or right chamber, with each chamber partitioned by a door. The experiment was divided into three phases. The test mouse was allowed to explore all three chambers freely for 10 min for habituation in the first phase. The second phase is the social interaction test. An age-matched novel mouse was put into a cage and a nonsocial novel object was put into another cage. The test mouse was gently put into the center and allowed to explore the three chambers for a 10-min session. Following the 10-min session, in the third phase, the nonsocial novel object was replaced by another novel mouse, and the test mouse was allowed to freely explore for 10 min. Time spent in each chamber was measured.

#### Open-field test

Locomotor activity and anxiety were measured using the open-field test. The test mouse was gently put into the open-field apparatus (72 cm by 72 cm by 40 cm) and then allowed to freely explore the area for 10 min. The total travel distance and the time spent in the center area (36 cm by 36 cm) were recorded.

#### Y-maze test

The Y-maze could be used to investigate cognitive ability and spatial working memory. This apparatus consisted of three radial arms positioned at equal angles (120°). At the beginning of trials, the test mouse was placed at the center area and allowed to freely move through the maze for 8 min. The efficient behavior was defined as the entry into all three arms on consecutive choices in overlapping triple sets.

#### Elevated-plus maze

Anxiety was measured using the elevated-plus maze test. The elevated-plus maze consisted of two open arms and two closed arms located 50 cm above the ground. The test mouse was put into the center of the maze. Then, time spent and distance traveled in different arms of 6 min of free exploration were measured.

#### Light/dark transition test

Anxiety was also measured using the light/dark transition test. The apparatus consisted of two sections: light compartment and dark compartment. Each test mouse was placed in the dark compartment and allowed to move freely for 10 min. The distance traveled and time spent in the light compartment were recorded.

#### *Repetitive behavior of grooming*, *digging, and rearing*

The test mouse was allowed to freely explore in the new empty cage for 10 min after 10 min of habituation. Mice exhibit spontaneous motor stereotypies, including self-grooming, digging, and rearing. The behavior was double-blind assessed by an investigator watching video recordings.

#### Marble-burying test

The marble-burying test was conducted to detect repetitive behaviors (*50*). The test cage was filled with 3 to 4 cm of corncob bedding, and 20 glass toy marbles were gently placed in a 5 × 4 pattern of the cage. Then, the test mouse was allowed to explore for 30 min. A marble was considered buried if at least two-thirds of its surface was covered by bedding.

### Immunostaining of brain slices

The day of vaginal plugging was designated as 0.5 (E0.5). For measuring cortical lamination, embryos were harvested at E18.5. For progenitor proliferation analyses, EdU (50 mg/kg) was injected intraperitoneally 30 min before dissection at E15.5. To induce stress in embryo, we applied AS (4 mg/g) to the mice by intragastric administration for 24 hours. Embryos were fixed by immersion in 4% PFA overnight. Then, 15% followed by 30% sucrose was used for dehydration of the brain. Immunostaining was performed on 20-μm frozen tissue sections using Leica CM 1950. The prepared brain slices were put into a 60°C dryer for 1 hour. Frozen sections were washed three times with 1× PBS for 10 min and then blocked with blocking buffer (1× PBS, 3% BSA, 10% FBS, and 0.2% Triton) for 1 hour at room temperature. Sections were incubated overnight at 4°C with primary antibodies. After washing three times with 1× PBS, secondary antibodies were applied to sections for 1 hour at room temperature. EdU detection was performed after immunostaining according to the protocol of a Click-it EdU Alexa 555 imaging kit (A10338). Sections were stained with DAPI for 1 min. Because of the morphological abnormality of *Ubap2l*-deficient mouse cortices, thalamic landmarks were used to match sections of different genotypes. Immunofluorescence images were obtained by a Leica THUNDER microscope and analyzed in ImageJ software.

### Functional enrichment analysis of SG genes

The 843 SG genes (including 262 SG assembly regulators) and 26 core SG components were collected from the union and intersection of three previously published works, respectively (*13*, *17*, *18*) (fig. S6A and table S3). The 361 well-curated high-confidence ASD genes were culled from the SFARI Gene database (https://gene.sfari.org/). Only high-confidence genes with a gene score of 1 or S were included. The 827 well-curated developmental disorder genes were pulled from the DDG2P database (www.deciphergenomics.org/ddd/ddgenes). Only genes with definitive, strong, and “both RD (relevant disease) and IF (incidental finding)” evidence and monoallelic mode were included. Note that “both RD and IF” show genes that are plausibly associated with both the RD and another disease that represents an IF, which highlights for clinical review genotypes (table S4). “FMRP binding targets” are genes encoding transcripts that bind to FMRP, which are identified by Darnell *et al.* (*28*). “CSDE1 binding targets” are genes encoding transcripts that bind to CSDE1, which are identified by Guo *et al.* and Wurth *et al.* (*26*, *29*). “RBFOX binding targets” are genes encoding transcripts that bind to RBFOX, which are identified by Weyn-Vanhentenryck *et al.* (*27*). “CHD8 binding targets” are genes encoding transcripts that bind to CHD8, which are identified by Cotney *et al.* (*30*) and Subtil-Rodriguez *et al.* (*31*). Enrichment analysis of SG genes in NDD genes and NDD-related gene sets was performed using a binomial distribution test based on a genome-wide background (19,982 genes).

### DNMs in SG genes and burden analysis

De novo single-nucleotide and indel variants in the coding region of the 843 SG genes were identified from 9228 patients with ASD and 31,625 ID/DD individuals from 26 whole-exome/genome sequencing studies (tables S6 and S7). We excluded the potential duplicated samples with the same identifier or carrying the same variant(s) if studies originated from the related cohorts [e.g., SSC (Simons Simplex Collection), ASC (Autism Sequencing Consortium), or MSSNG]. All variants were annotated by ANNOVAR (*51*). Variants were mainly divided into four categories [LGD (stopgain, splice site, and frameshift), missense, non-frameshift, and synonymous].

Burden analysis of SG genes with de novo coding variants was performed using two probabilistic models with default settings: CH model and DenovolyzeR. Briefly, we derived the expected number of de novo events in a given population based on the mutability of a gene and the number of probands sequenced and compared the observed number of DNMs against expectation using a Poisson framework (DenovolyzeR) or binormal model (CH model) for all SG components (*32*, *33*). In detail, the rates of de novo events in DenovolyzeR were calculated on the basis of the context of triplet nucleotide, and the CH model used the rates of base substitution from chimpanzee to human as DNM rates (*32*, *52*). For conservative statistics, the expected number of DNMs per individual is 1.8. Correction for multiple tests was performed using the Benjamini-Hochberg method considering 843 genes and two statistical models (times for correction: 843 × 2).

### PPI and GCE networks

A PPI network of 26 core SG genes was built by STRING (v11), and only physical PPIs were considered on the basis of the SG features (*53*). For a GCE network, RNA sequencing data (unit: reads per kilobase per million mapped reads) at different developmental stages (from eight postconceptional weeks to 40 years) for cortex development were obtained from BrainSpan (*54*). Spearman correlation analysis was carried out for gene pairs of core SG genes, and gene pairs with |*R*| > 0.7 were used to establish the GCE network. Cytoscape 3.7.1 was applied for network visualization and calculating the centrality of nodes (closeness centrality and betweenness centrality) (*55*).

### Network enrichment analysis

The enrichment of targeted subnetworks was analyzed by comparing the connectivity of an observed subnetwork (e.g., core SG subnetwork) with the distribution of connectivity of a background network (e.g., all SG subnetworks). The distribution of network connectivity was random sampling 10,000 subnetworks from a background network. Network connectivity represents the average of node connectivity.

## Supplementary Materials

### This PDF file includes:

Figs. S1 to S9 Supplementary Notes References

### Other Supplementary Material for this manuscript includes the following:

Data S1 to S8

Correction 19 September 2022:

Due to a production error, a supplemental data file was not included with the original publication of this paper. This file has now been included, and the supplemental PDF has been updated with captions for this data. The original version is available here:

## Notes

View/request a protocol for this paper from *Bio-protocol*.
